# A norovirus intervariant GII.4 recombinant in Victoria, Australia, June 2016: the next epidemic variant?

**DOI:** 10.2807/1560-7917.ES.2016.21.39.30353

**Published:** 2016-09-29

**Authors:** Leesa Bruggink, Michael Catton, John Marshall

**Affiliations:** 1 Victorian Infectious Diseases Reference Laboratory, Royal Melbourne Hospital, The Peter Doherty Institute for Infection and Immunity, Melbourne, Victoria, Australia

**Keywords:** norovirus, epidemic, variant, pandemic, recombination, emergence

## Abstract

A norovirus recombinant GII.P4_NewOrleans_2009/GII.4_Sydney_2012 was first detected in Victoria, Australia, in August 2015 at low frequency, and then re-emerged in June 2016, having undergone genetic changes. Analysis of 14 years’ surveillance data from Victoria suggests a typical delay of two to seven months between first detection of a new variant and occurrence of a subsequent epidemic linked to that variant. We consider that the current recombinant strain has the potential to become a pandemic variant.

This study reports the emergence of a GII.4 intervariant recombinant of GII.P4_NewOrleans_2009 (ORF1) with GII.4_Sydney_2012 (ORF2). This new recombinant – first detected in Victoria, Australia, in August 2015, then re-emerged, with genetic changes, in June 2016 – has been the causative agent in the majority of norovirus gastroenteritis outbreaks in Victoria since its remergence. It is proposed that the pattern of emergence of this strain renders it a potential candidate to become the next GII.4 pandemic variant.

Norovirus strains can be highly adaptable, escaping herd immunity and thus continuing to infect the community over long periods. There is evidence that the emergence of new global epidemic variants and their subsequent global spread is rapid, with almost simultaneous detection worldwide [1-4]. GII.4 noroviruses cause ca 70–80% of all human norovirus-associated gastroenteritis worldwide [5] and pandemics of norovirus infection occurred in 1996, 2002, 2004, 2006, 2009 and 2012, all caused by the emergence of new GII.4 variants [1,6]. No new pandemic strain has emerged since the GII.4_Sydney_2012 variant [5], which has been the predominant strain in Victoria, Australia, since its emergence in 2012, although there is a report [7] of an altered form of the Sydney_2012 variant (referred to as the Sydney_2015 variant) detected over the past 12 months in the United States. The report [7] appears to be based only on partial capsid sequence and it is unclear whether Sydney_2015 is the GII.P4_NewOrleans_2009/GII.4_Sydney_2012 recombinant.

## Origin of faecal material for norovirus testing

The Victorian Infectious Diseases Reference Laboratory (VIDRL) is the main public health laboratory for viral identification in the State of Victoria, Australia. Faecal specimens collected from gastroenteritis outbreaks are routinely sent to VIDRL for norovirus testing [8]. An outbreak of gastroenteritis was defined as an incident, apparently associated with an event or location, in which four or more individuals had symptoms of gastroenteritis. During 14 years (2002–15), VIDRL has received a mean of 1,296 faecal specimens per year from gastroenteritis outbreaks. These specimens are from a mean of 273 outbreaks per year, with norovirus detected in a mean of 177 outbreaks per year.

## Detection of norovirus and sequencing protocols

Faecal specimens were processed as described previously [8] and then tested by an ORF1 reverse transcription (RT)-PCR that detects both GI and GII norovirus [8]. Additionally, a GII ORF2 RT-PCR was also performed on one specimen from each gastroenteritis outbreak [8]. Where ORF1 and ORF2 testing provided different genotypes, an ORF1-ORF2 bridging PCR was performed to try and confirm recombination status [9]. Full capsid sequence was obtained using primers developed by Kim et al. [10].

**Figure 1:**
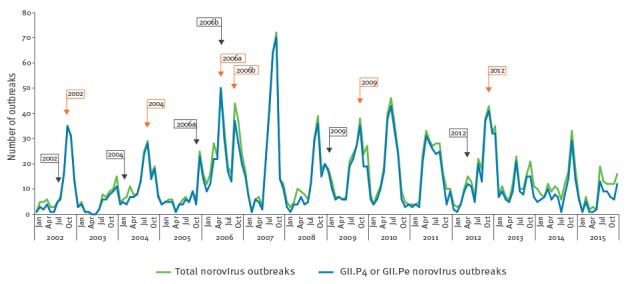
Norovirus gastroenteritis outbreak periodicity, Victoria, Australia, 2002–15 (n = 2,473)

Nucleotide sequencing and phylogenetic analysis were carried out as described previously [11] using the software MacVector v15.0, Phylip v3.695 and FigTree v1.4.2. Genotype analysis also made use of the norovirus genotyping tool [12,13]. Information presented on norovirus gastroenteritis outbreak periodicity during 2002 to 2015 (Figure 1) and the associated GII.4 variant information (Figure 1, Table) made use of sequencing information from previous studies in our laboratory [11,14] as well as inclusion of novel data.

Monthly number of norovirus gastroenteritis outbreaks in the State of Victoria, Australia, from 2002 to 2015 (based on ORF1 reverse transcription (RT)-PCR data). The total number of norovirus outbreaks is shown, as well as the outbreaks where GII.P4 or GII.Pe (ORF1) was detected. It is notable that in most years an epidemic peak occurred in the latter half of the year (spring–summer in Australia). An epidemic ‘peak’ was considered to be three consecutive months of the highest number of norovirus gastroenteritis outbreaks in a calendar year, except in 2006 where there were two epidemic peaks of similar size [14]. Black arrows indicate the first detection of a new epidemic GII.4 variant (Farmington_Hills_2002, Hunter_2004, Yerseke_2006a, Den Haag_2006b, NewOrleans_2009 and Sydney_2012). Orange arrows indicate the first epidemic peak caused by a new epidemic GII.4 variant. This graph makes use of sequencing information from previous studies in our laboratory [11,14] as well as inclusion of novel data.

**Table:** Norovirus GII.4 variants that emerged and led to gastroenteritis epidemics in Victoria, Australia, 2002–15^a^

Norovirus GII.4 variant	Month and year of first detection	First epidemic peak^b^	Delay^c^ in months
Farmington_Hills_2002	July 2002	September–November 2002	2
Hunter_2004	February 2004	August–October 2004	6
Yerseke_2006a	December 2005	May–July 2006	5
Den Haag_2006b	June 2006	October–December 2006	4
NewOrleans_2009	January 2009	August–October 2009	7
Sydney_2012	May 2012	October–December 2012	5

^a^ This table makes use of sequencing information from previous studies in our laboratory [11,14] as well as inclusion of novel data.

^b^ An epidemic ‘peak’ was considered to be three consecutive months of the highest number of norovirus gastroenteritis outbreaks in a calendar year, except in 2006 where there were two epidemic peaks of similar size [14].

^c^ Time delay from first detection to the beginning of the first epidemic peak.

## Norovirus periodicity and GII.4 variants

Although norovirus is detected throughout the year, there is generally one norovirus epidemic peak of outbreaks per calendar year. An epidemic ‘peak’ was considered to be three consecutive months of the highest number of norovirus gastroenteritis outbreaks in a calendar year, except in 2006 where there were two epidemic peaks of similar size [14]. During 2002 to 2015 there was a single major yearly epidemic peak, except in 2006 when there were two (Figure 1). Norovirus epidemics are generally linked to a GII.4 genotypic variant, with a new variant typically emerging every two to three years at a time point between annual peaks of norovirus detection. In 2006, there was an almost simultaneous emergence of two new epidemic GII.4 norovirus variants, Yerseke_2006a and Den Haag_2006b, with the Den Haag_2006b variant emerging during the Yerseke_2006a epidemic peak.

The emergence of a new norovirus epidemic variant results in an epidemic peak in the number of outbreaks that are predominantly due to the new variant, usually in the year that it emerges and then in a number of subsequent years, until a successor variant emerges. The Yerseke_2006a variant only caused the first epidemic peak in 2006 and was immediately replaced as the predominant variant by the Den Haag_2006b variant. Analysis of the data from all six variants that emerged during 2002 to 2015 shows that there was a delay of between two and seven months from the first detection of a new variant to the time of the first epidemic peak linked to that variant (Figure 1, Table).

**Figure 2:**
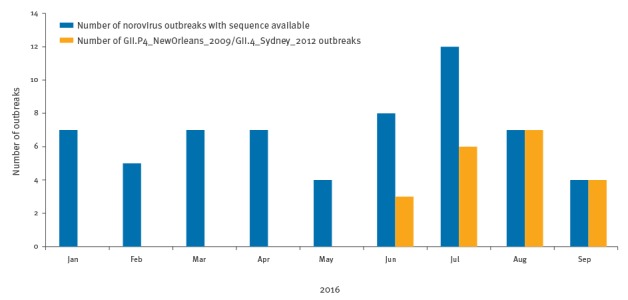
Norovirus gastroenteritis outbreaks in Victoria, Australia, 2016^a^ (n = 61)

A recently emerged new variant is the GII.4 intervariant recombinant of GII.P4_NewOrleans_2009 (ORF1) with GII.4_Sydney_2012 (ORF2). This recombinant was first detected in Victoria in August 2015 and was only detected at low levels in late 2015 (three of 64 outbreaks in August to December 2015). This strain then remained undetected for five months (January to May 2016) and re-emerged in mid-June 2016 (Figure 2). Since its re-emergence in June 2016, this recombinant strain has caused the majority of the norovirus gastroenteritis outbreaks detected in Victoria (6/12 outbreaks in July, 7/7 outbreaks in August and 4/4 outbreaks in September (up to 20 September)). An ORF1-ORF2 sequence of the first detected recombinant strain from 2015 has been lodged in GenBank under accession number KX064756.

Monthly number of norovirus gastroenteritis outbreaks in the state of Victoria, Australia, 2016 (based on ORF1 reverse transcription (RT)-PCR and sequencing data). The total number of norovirus outbreaks with sequence data available is shown, as well as the number of outbreaks where the GII.P4_NewOrleans_2009/GII.4_Sydney_2012 recombinant was detected.

^a^ Data up to 20 September 2016.

## Phylogenetic analysis

**Figure 3:**
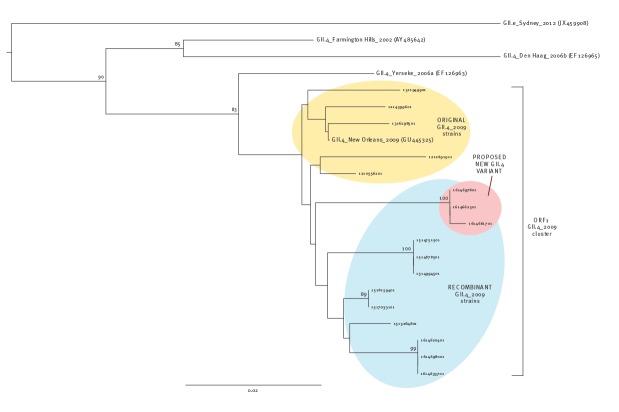
Phylogenetic tree of an ORF1 fragment of norovirus GII.4 strains

**Figure 4:**
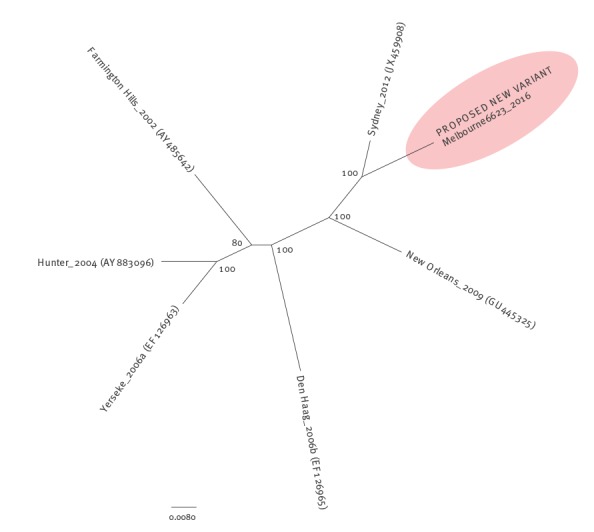
Phylogenetic tree of the full capsid sequence of norovirus GII.4 variant reference strains

The recombinant strain differs from the original parent strains in both ORF1 (Figure 3) and ORF2 (Figure 4). Furthermore, the recombinant strain has undergone some minor alterations, making some of the 2016 strains cluster separately on a phylogenetic tree from earlier 2015 and 2016 recombinant strains (Figure 3).

DNA (dist) Kimura phylogenetic tree of a 440 bp norovirus ORF1 fragment of norovirus GII.4 strains corresponding to positions 4,484–4,923 of reference strain GII.4_Sydney_2012 (JX459908). Yellow highlights the branch containing the original strain GII.4_NewOrleans_2009 (GU445325) as well as representative specimens of that genotype from 2012 and 2013. Blue highlights the branch containing the recombinant GII.P4_NewOrleans_2009/GII.4_Sydney_2012 with specimens from 2015 and 2016. Red highlights the branch containing the proposed new variant that has altered slightly and is branching away from the recombinant form. The figures on the branches represent bootstrap values (%) after resampling 1,000 datasets. Only bootstrap values ≥ 70% are shown. The scale marker represents substitutions per site.

DNA (dist) Kimura phylogenetic tree of GII.4 norovirus full capsid sequence of norovirus GII.4 variant reference strains corresponding to positions 5,085–6,707 (1,623bp) of reference strain GII.4_Sydney_2012 (JX459908). Red highlights the branch containing the potential new variant that has altered slightly and is branching away from the parent strain GII.4_Sydney_2012 (JX459908). The figures on the branches represent bootstrap values (%) after resampling 1,000 datasets. Only bootstrap values ≥ 70% are shown. The scale marker represents substitutions per site.

A phylogenetic tree of the full capsid sequence (Figure 4) shows that the new strain differs from all previous major epidemic variants. We consider that this altered recombinant strain holds the potential to be a new epidemic variant.

Examination of the percentage nucleotide similarity of full capsid sequence from past epidemic variants (2002 AY485642, 2004 AY883096, 2006a EF126963, 2006b EF126965, 2009 GU445325, 2012 JX459908) [6] demonstrates that the variants share 91.4–96.5% similarity. The full capsid sequence of one of the first altered recombinant strains from this study (GII.4/Melbourne6623/2016/AUS) detected in June 2016 has been lodged in GenBank (KX767083). The nucleotide similarity of this strain compared with its closest counterpart, Sydney_2012 (JX459908) is 96.3%, which is consistent with the range given above.

## GII.4 hypervariable epitopes

**Figure 5:**
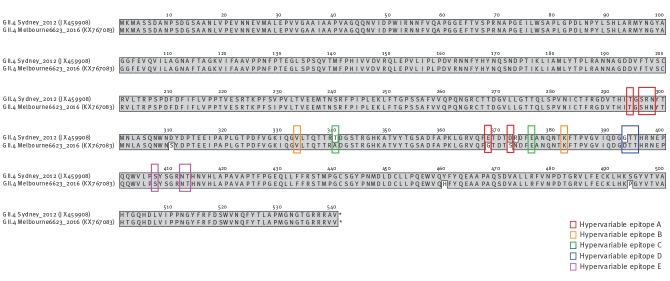
Amino acid alignment of the full capsid protein of the GII.4_Sydney_2012 norovirus reference strain and the proposed new variant detected in June 2016 in Victoria, Australia

A fundamental study by Lindesmith et al. [15] proposed five hypervariable epitopes (A to E) for GII.4 noroviruses that appear to evolve over time and drive antigenic change, allowing the emergence of new strains that can evade the population’s immune response. Amino acid alignment of the full capsid protein of the proposed new variant with that of its closest counterpart, Sydney_2012 (JX459908), shows that of the five epitopes proposed by Lindesmith et al. [15], three have undergone change in the proposed new variant (Figure 5). The three altered epitopes are A, C and D.

Amino acid alignment of norovirus full capsid protein of GII.4_Sydney_2012 (JX459908) and the proposed new variant GII.4_Melbourne6623_2016 (KX767083) from this study. Hypervariable epitopes A to E [15] are outlined.

## Discussion

The classification of new GII.4 variants is based both on phylogenetic analysis and on the presence of the variant becoming epidemic in at least two distinct geographical locations [6]. The recombination of two past epidemic variants (GII.P4_NewOrleans_2009 and GII.4_Sydney_2012), followed by additional evolutionary changes in both ORF1 and ORF2, provides the new strain with genetic novelty. In particular, changes in three of the five hypervariable epitopes proposed by Lindesmith et al. [15] illustrate that the strain has changed at sites critical for the evolution of the virus. The strain has already become predominant between norovirus seasons in outbreaks in Victoria, Australia, over the past three months (July to September). On this basis, we propose it as a candidate new epidemic strain. If past trends are followed, then it would have the potential to also predominate in other parts of the world.

When 14 years of norovirus gastroenteritis outbreak incidence data are analysed for epidemic GII.4 variants, it can be seen that between two and seven months typically pass between first detection of the variant and the subsequent epidemic. If this new recombinant has undergone enough change to escape herd immunity and become the next epidemic variant, then from its first detection in Victoria in mid-June 2016, the expected epidemic could be any time between mid-August 2016 and January 2017. As the emergence of new global epidemic variants and their subsequent spread is rapid, and they are detected almost simultaneously worldwide [1-4], the rest of the world could also undergo a norovirus epidemic within a similar time frame.

A key observation in this report is the delay between the first appearance of an epidemic norovirus strain and the subsequent epidemic involving that strain. This observation accords well with the recent findings of Allen et al. [16], who suggest that pandemic strains may circulate at low levels in the community up to several years before their global spread. The concept of delay in norovirus epidemic variant circulation is still poorly understood and clearly requires further investigation.
